# Steam turbine power prediction based on encode-decoder framework guided by the condenser vacuum degree

**DOI:** 10.1371/journal.pone.0275998

**Published:** 2022-10-27

**Authors:** Yanning Lu, Yanzheng Xiang, Bo Chen, Haiyang Zhu, Junfeng Yue, Yawei Jin, Pengfei He, Yibo Zhao, Yingjie Zhu, Jiasheng Si, Deyu Zhou

**Affiliations:** 1 Power Engineering Center, Jiangsu Frontier Electric Technology Co., Ltd., Nanjing, Jiangsu, China; 2 School of Computer Science and Engineering, Key Laboratory of Computer Network and Information Integration, Ministry of Education, Southeast University, Nanjing, Jiangsu, China; Hanyang University, KOREA, REPUBLIC OF

## Abstract

The steam turbine is one of the major pieces of equipment in thermal power plants. It is crucial to predict its output accurately. However, because of its complex coupling relationships with other equipment, it is still a challenging task. Previous methods mainly focus on the operation of the steam turbine individually while ignoring the coupling relationship with the condenser, which we believe is crucial for the prediction. Therefore, in this paper, to explore the coupling relationship between steam turbine and condenser, we propose a novel approach for steam turbine power prediction based on the encode-decoder framework guided by the condenser vacuum degree (CVD-EDF). In specific, the historical information within condenser operation conditions data is encoded using a long-short term memory network. Moreover, a connection module consisting of an attention mechanism and a convolutional neural network is incorporated to capture the local and global information in the encoder. The steam turbine power is predicted based on all the information. In this way, the coupling relationship between the condenser and the steam turbine is fully explored. Abundant experiments are conducted on real data from the power plant. The experimental results show that our proposed CVD-EDF achieves great improvements over several competitive methods. our method improves by 32.2% and 37.0% in terms of RMSE and MAE by comparing the LSTM at one-minute intervals.

## Introduction

A stable electricity supply is an important guarantee for effective production. Thermal power generation is currently one of the most important power generation ways in the world. A complete thermal power generation system contains different equipment with different functions (e.g., circulating water pumps, condensers, steam turbines and other equipment, where the steam turbine is the power generation equipment and the condenser is the main auxiliary equipment). [1] has pointed out that accurate power forecasting is crucial for steam turbine control which is a complex and challenging task.

There exist many approaches for output power forecasting of turbines. Some approaches adopt machine learning methods, such as regression-based methods, to predict the output power of turbines. Furthermore, many neural network-based methods have been used to exploit the features of turbine operating data and predict the output power of turbines more accurately.

However, a notable drawback of the methods mentioned above is that they only focus on the output power prediction based on the turbine information individually while ignoring the correlation with the rest equipment of the power generation system. For example, they ignore the coupling relationship between the condenser (the front-end equipment of the steam turbine) and the steam turbine, which is contradictory to the practical scenario because some of the input factors are unavailable in reality (e.g., temporally condenser vacuum). In this paper, we explore the prediction of the steam turbine output power by introducing the coupling relationship with the condenser. A natural intermediary for coupling the condenser and the steam turbine is the condenser vacuum degree (i.e., an indicator reflecting the working status of the condenser and an important metric for the operation of the thermometric generating set.), which is a key factor for predicting the output power of the steam turbine. However, as an intermediate factor between the condenser and the steam turbine, the condenser vacuum degree varies dynamically based on the time and the condition status of the equipment [2–4], which brings difficulty in accurate modelling the vacuum degree temporally. In addition, since the different types of input variables for the condenser and the steam turbine, it is challenging to jointly model these two pieces of equipment by introducing the vacuum degree information into the output power prediction.

To overcome the above issues, in this paper, we propose an **C**ondenser **V**acuum **D**egree guided approach based on **E**ncoder-**D**ecoder **F**ramework for steam turbine output power prediction (CVD-EDF). We model the condenser and steam turbine at the encoder and decoder, respectively. The encoder predicts the condenser vacuum degree dynamically and the decoder predicts the steam turbine output power at the target moment. In specific, we adopt the multi-layer LSTM as the basic architecture of the encoder and the decoder. Moreover, a connection module consisting of an attention mechanism and a convolutional neural network (CNN) is proposed to capture the local and global information within the encoder. All the information is further introduced into the decoder to predict the steam turbine output power at the target moment.

In summary, the main contributions of this paper are listed as follows:

- A novel condenser vacuum degree guided approach based on the Encoder-Decoder framework for steam turbine power prediction is proposed. To the best of our knowledge, our work is the first attempt to take into account the coupling relationship between the steam turbine and the condenser when predicting the steam turbine output power.
- Experimental results on the real data from the power plant show that our proposed CVD-EDF outperforms several competitive baselines. It achieves an improvement of 32.2% RMSE and an improvement of 37.0% MAE by comparing the LSTM at one-minute intervals.

## Related work

### Encoder-Decoder framework

The Encoder-Decoder framework is popular in artificial intelligence which consists of an encoder and a decoder. The encoder is a neural structure that extracts features from raw inputs and passes them to the decoder. The decoder is another neural structure that incorporates the features from the encoder and makes decisions for the task. In the beginning, the framework is widely used in the field of signal processing because of its ability to compress dimensions. [5] adopts the auto-encoder [6] for bio-signals compression and [7] propose to use convolutional auto-encoder for ECG signals compression. The auto-encoder is based on the encoder-decoder framework and it can learn a latent space in an unsupervised way. Later, the encoder-decoder framework became popular in the field of natural language processing [8–11], using it to process two tasks simultaneously at the encoder and decoder. [12] first applies it to neural machine translation. The framework is suitable for tasks that generate a sequential output and it can also be applied to other areas such as computer vision and speech processing [13–15]. However, there is no approach that adopts the Encoder-Decoder framework for output power prediction of the steam turbine. In this paper, we first apply the Encoder-Decoder framework to model the thermal power generation system. The reason is that we can process two tasks simultaneously in the encoder and decoder and introduce information from the encoder into the decoder in a flexible way. The condenser and the steam turbine are modelled in the encoder and decoder respectively.

### Output power prediction of turbines

In general, the methods of predicting the output power of turbines can be divided into two categories according to their basic techniques and methodologies, i.e., machine learning approaches and deep learning approaches. For machine learning approaches, [16] adopts two non-parametric techniques based on the tilting method and monotonic spline regression to predict the power of the wind turbine. [17] proposes a non-linear regression model for wind turbine power curve approximation. Polynomial regression and exponential power curves have also been applied for output power prediction [18–20]. With the development of deep learning, many neural network-based methods are proposed for steam turbine power prediction. Artificial neural network (ANN) models representing the real power plant have been introduced into the steam turbine power prediction task [21, 22]. [23] adopts a long short-term memory network (LSTM) to forecast the wind turbine power and further uses the Gaussian mixture model (GMM) to analyze the error distribution characteristics of short-term wind turbine power forecasting. [24] proposes to adopt a neural network to establish accurate numerical simulators of the power plant units. However, all the approaches mentioned above have limitations because they only consider the information of the turbine and largely ignore the correlation with the rest equipment of the power generation system.

[25] takes into account not only the turbine but also the boiler when predicting the output power. They propose to utilize two ANN models, one for the boiler and one for the turbine, which are integrated to predict the power output from a coal-fired plant. However, our goal in this paper is to explore an approach from a different perspective, i.e., exploring the prediction of the steam turbine output power by introducing the coupling relationship with the condenser. [26] propose a novel hybrid framework for hotspot prediction which also contains LSTM and CNN, but this framework is markedly different from ours, which is based on an Encoder-Decoder framework. And we are the first to adopt the Encoder-Decoder framework for output power prediction of the steam turbine.

## Preliminary

### LSTM

Long short-term memory network (LSTM) [27] is a Recurrent Neural Networks (RNN) [28, 29] architecture which is widely used in output power prediction of the steam turbine. It has significant advantages in processing time series data, which leverages time series dependencies between data. A standard RNN cannot bridge more than 5-10 time steps [30] and the reason is that the back-propagating error signal tends to grow or shrink with each time step [31].

**Fig 1:**
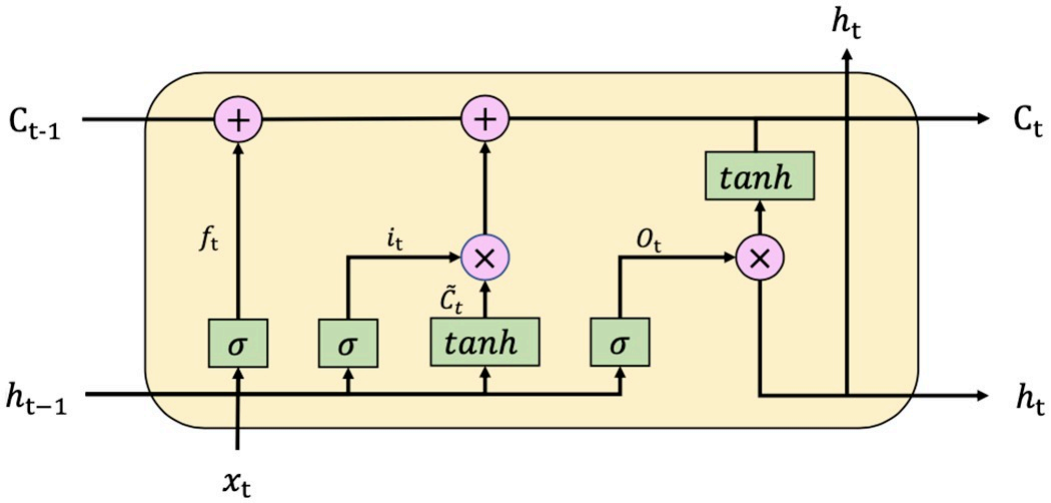
The architecture of LSTM.

LSTM is designed to handle long-time dependencies and the architecture of LSTM is shown in Fig 1. A common LSTM cell consists of a cell, an input gate, an output gate, and a forget gate. The three gates are composed of a sigmoid neural net layer and a pointwise multiplication operation. The outputs of gates are numbers range [0, 1]. They control the input, output and forgetting of past information of the cell respectively. These three gates regulate the flow of information into and out of the cell. Due to the structure of LSTM, it is able to access information at a more distant step.

At time step *t*, the input of LSTM is *x*_*t*_ and the hidden states and the cell states at time step *t* − 1 are *h*_*t*−1_, *c*_*t*−1_ respectively. The forget gate *f*_*t*_ decides what information should be discarded and it is calculated as follows:

$$\begin{array}{c} f_{t}=\sigma \left(W_{f}.\left[h_{t-1},x_{t}\right]+b_{f}\right) \end{array}$$ (1)

Where *W*_*f*_ is a trainable parameter and *b*_*f*_ is a bias vector. The input gate *i*_*t*_ determines what information should be stored in the cell states, and the new candidate value ${\tilde{c}}_{t}$ is obtained:

$$\begin{array}{c} i_{t}=\sigma \left(W_{i}.\left[h_{t-1},x_{t}\right]+b_{i}\right) \end{array}$$ (2)

$$\begin{array}{c} {\tilde{c}}_{t}=tanh(W_{c}.\left[h_{t-1},x_{t}+b_{c}\right) \end{array}$$ (3)

Where *W*_*i*_, *W*_*c*_ are trainable parameters and *b*_*i*_, *b*_*c*_ are bias vectors. The cell states *c*_*t*_ at time step t is calculated as follows:

$$\begin{array}{c} c_{t}=f_{i}*c_{t-1}+i_{t}*{\tilde{c}}_{t} \end{array}$$ (4)

And the output gate *o*_*t*_ determines what is going to be output, and the output *h*_*t*_ can be obtained:

$$\begin{array}{c} o_{t}=\sigma \left(W_{o}.\left[h_{t-1},x_{t}\right]+b_{o}\right) \end{array}$$ (5)

$$\begin{array}{c} h_{t}=o_{t}*tanh\left(c_{t}\right) \end{array}$$ (6)

Where *W*_*o*_ is a trainable parameter and *b*_*o*_ is a bias vector. Following previous research, we adopt LSTM as the basic component for constructing our model in this paper.

## Method

**Fig 2:**
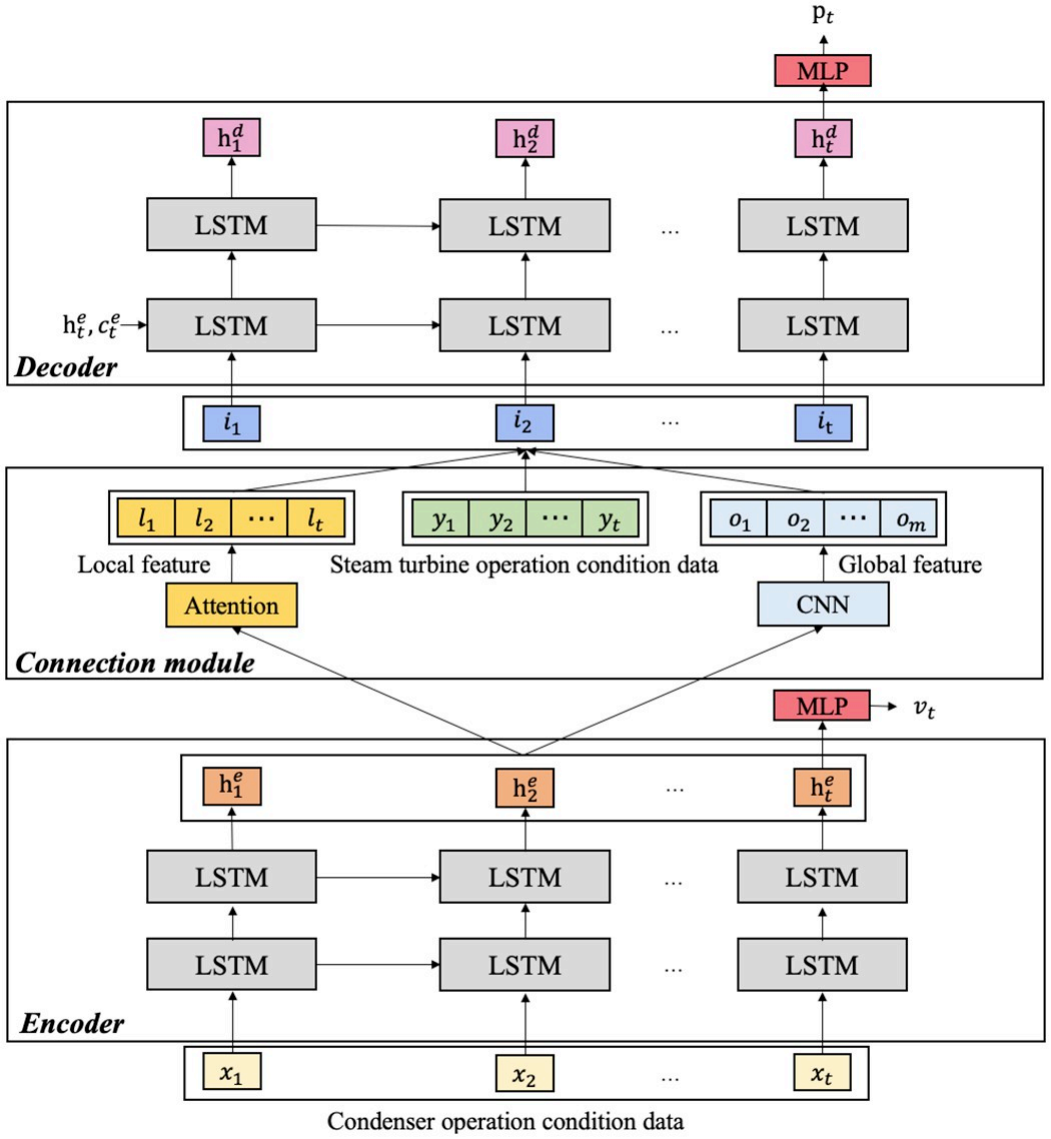
The overview architecture of the proposed CVD-EDF.

In this paper, we explore the coupling relationship between the steam turbine and the condenser and propose a novel approach for steam turbine power prediction based on the encode-decoder framework guided by the condenser vacuum degree (CVD-EDF). We introduce the proposed CVD-EDF in detail in this section. The model architecture is shown in Fig 2, which consists of three parts:

1. Encoder: A LSTM is adopted to capture the historical information of the condenser operating conditions data, and the condenser vacuum degree of the target moment is predicted through a multi-layer perceptron network (MLP).
2. Connection module: An attention mechanism and a convolutional neural network (CNN) [32] are proposed to capture the local and global features of the hidden states from the encoder respectively at each step of the decoding process.
3. Decoder: The local, global features and the steam turbine operating condition data are concatenated as the input of the decoder. In this way, the information of the condenser vacuum is introduced into the decoder. Then, the history information of the steam turbine operating conditions data is captured by another LSTM. The initial hidden states and cell states of the decoder LSTM are initialled with the last hidden states and cell states of the encoder LSTM. The output power of the steam turbine at the target moment is predicted by fusing various information.

### Encoder

In the encoder part, the condenser is modelled. The input of the encoder *X* = [*x*_1_, …, *x*_*i*_, …, *x*_*t*_] is the historical data sequence of the condenser operating conditions, where *t* is the length of the sequence. The encoder extracts features from *X* and predicts the vacuum degree at the target time step.

We apply a multi-layer LSTM as the encoder to extract time series patterns from the input. The initial hidden states $h_{0}^{e}$ and cell states $c_{0}^{e}$ of the encoder LSTM are initialized to zero. The hidden states $H^{e}=\left[h_{1}^{e},\ldots ,h_{i}^{e},\ldots ,h_{t}^{e}\right]$ and cell states $C^{e}=\left[c_{1}^{e},\ldots ,c_{i}^{e},\ldots ,c_{t}^{e}\right]$ of the last layer of the encoder can be obtained, mapping historical data from the original space to feature space:

$$\begin{array}{c} H^{e},C^{e}=LSTM\left(X,H_{t-1}^{e},C_{t-1}^{e}\right) \end{array}$$ (7)

The condenser vacuum degree *v*_*t*_ at timestep t can be calculated via:

$$\begin{array}{c} v_{t}=W_{2}ReLU\left(W_{1}h_{t}^{e}\right) \end{array}$$ (8)

Where *W*_1_ and *W*_2_ are trainable parameters, and $h_{t}^{e}$ is the last encoder hidden state at timestep t. *ReLU* denotes the ReLU [33] activate function.

### Connection module

The Connection module is proposed to capture the vacuum degree information from the encoder and pass it to the decoder. An attention mechanism and a CNN are adopted to capture the local and global features from the encoder part. The extracted features will be used as input to the decoder.

For local feature *l*_*i*_ at time step *i*, We can explicitly determine the semantic relevance between the decoder hidden state $h_{i-1}^{d}$ (Eq 15) at time step *i* − 1 and *H*^*e*^ by calculating the dot product:

$$\begin{array}{c} A_{l}=softmax\left(h_{i-1}^{d}H^{eT}\right) \end{array}$$ (9)

The local feature *l*_*i*_ at time step *i* is calculated as follows:

$$\begin{array}{c} l_{i}=A_{l}H^{e} \end{array}$$ (10)

For global feature *G*, we adopt a 1-D convolution to perform feature mapping on *H*^*e*^:

$$\begin{array}{c} h_{n}^{i}=f\left(w_{i}H_{n:n+s-1}^{e}\right) \end{array}$$ (11)

$$\begin{array}{c} o_{i}=max\left(h_{1:t-s+1}^{i}\right) \end{array}$$ (12)

Where *s* is the size of filters, *n* is the stride of convolution, *w*_*i*_ represents the parameters of the i-th filter and *f*(.) denotes the activation function. Then, max pooling is applied to reduce the dimensionality of the convolution output $h_{1:t-s+1}^{i}$. We concatenate the output of all filters to obtain the global feature *G*:

$$\begin{array}{c} G=[o_{1},o_{2},\ldots ,o_{m}] \end{array}$$ (13)

Where *m* is the number of filters.

### Decoder

In the decoder part, the steam turbine is modelled. To take into account the coupling relationship between the condenser and the steam turbine, the local and global features extracted by the connection module are used as additional inputs to the decoder. The decoder fuses the multiple features and predicts the output power at the target time step. The historical data sequence *Y* = [*y*_1_, …, *y*_*i*_, …, *y*_*t*_] of the steam turbine operating conditions is concatenated with the local features *L* = [*l*_1_, …, *l*_*i*_, …, *l*_*t*_] and the global features *G* and forming the input *I* = [*i*_1_, …, *i*_*i*_, …, *i*_*t*_] of the decoder:

$$\begin{array}{c} I=[L:Y:G] \end{array}$$ (14)

Another multi-layer LSTM is adopted as the decoder. The initial hidden states $h_{0}^{d}$ and cell states $c_{0}^{d}$ of the decoder are initialled with the last hidden states $h_{t}^{e}$ and cell states $c_{t}^{e}$ of the encoder LSTM. In this way, the condenser vacuum information can be introduced into the decoder. Then the hidden states $H^{d}=\left[h_{1}^{d},\ldots ,h_{i}^{d},\ldots ,h_{t}^{d}\right]$ and cell states $C^{d}=\left[c_{1}^{d},\ldots ,c_{i}^{d},\ldots ,c_{t}^{d}\right]$ of the last layer of the decoder can be obtained:

$$\begin{array}{c} H^{d},C^{d}=LSTM\left(I,H_{t-1}^{d},C_{t-1}^{d}\right) \end{array}$$ (15)

The steam turbine power *p*_*t*_ at timestep *t* can be calculated via:

$$\begin{array}{c} p_{t}=W_{4}ReLU\left(W_{3}h_{t}^{d}\right) \end{array}$$ (16)

Where *W*_3_ and *W*_4_ are trainable parameters, and $h_{t}^{d}$ is the last hidden state at timestep t.

### Training loss

We adopt the mean square error loss (MSE) to represent the error of the predicted condenser vacuum degree *v*_*t*_, the predicted steam turbine output power *p*_*t*_ with the real value:

$$\begin{array}{c} L_{1}=\frac{1}{N}\sum_{t=1}^{N}{\left(v_{t}-V_{t}\right)}^{2} \end{array}$$ (17)

$$\begin{array}{c} L_{2}=\frac{1}{N}\sum_{t=1}^{N}{\left(p_{t}-P_{t}\right)}^{2} \end{array}$$ (18)

where *P*_*t*_ and *V*_*t*_ represent the real condenser vacuum degree and the real steam turbine output power. *N* is the number of training samples. The training objective is to minimize the total loss L:

$$\begin{array}{c} L=L_{1}+L_{2} \end{array}$$ (19)

## Experiments

In this section, we evaluate the effectiveness of CVD-EDF by comparing it to other approaches and ablating several design choices in CVD-EDF to understand their contributions.

### Dataset

The data used for experiments are collected from a thermal power plant via sensors in Jiangsu Province of China, which range from July 1 to November 22, 2021, at a time interval of one minute.

For condenser operating conditions, the following real-time data needs to be collected: feedwater flow, circulating water inlet pressure, reheat steam temperature, reheat steam pressure, main steam temperature, main steam pressure, main steam flow rate, heat supply temperature, heat supply pressure, and heat supply flow rate. For steam turbine operating conditions, the following real-time data needs to be collected: supply line flow, supply line pressure, supply line temperature, reheat steam temperature, reheat steam pressure, main steam temperature and main steam pressure. Moreover, real-time data on the vacuum degree of the condenser and the output power of the steam turbine also needs to be collected.

As shown in Table 1, The amount of data is 208,677, and we divided them into the training set and test set, containing 180000 and 28677 samples respectively.

**Table 1:** Statistics of the dataset.

Dataset	Total	Training set	Test set
**Num**	208,677	180,000	28,677

### Experimental settings

The length of historical data sequence t is set to 10. For CVD-EDF, the number of layers of the LSTM encoder and LSTM decoder is 2, and the hidden state dimension is set to 64. The whole model is trained by the Adam optimizer [34] with a learning rate of 1e-3. The number of epochs is 40 and the mini-batch size of the input is set to 32. The number of parameters in the model is 104,210 and the hyper-parameters are chosen based on the evaluation results from the test set.

### Metrics

Root mean squared error and mean absolute error are adopted to evaluate the overall performance.

1. Root Mean Squared Error (RMSE): It is a standard way to measure a model in predicting quantitative data. *y*_*t*_ and ${\hat{y}}_{t}$ represent the ground truth and predicted value respectively, and RMSE is computed as follows:
  $$\begin{array}{c} RMSE=\sqrt{\frac{1}{N}\sum_{t=1}^{N}{\left(y_{t}-{\hat{y}}_{t}\right)}^{2}} \end{array}$$ (20)
2. Mean Absolute Error (MAE): It measures the average magnitude of the errors in a set of predictions:
  $$\begin{array}{c} MAE=\sum_{t=1}^{N}\left|y_{t}-{\hat{y}}_{t}\right| \end{array}$$ (21)

### Baselines

Some machine learning methods are chosen as the baselines, including several regression algorithms: Linear Regression, Ridge regression, LASSO regression, Elastic Net regression, Decision Tree regression and Xgboost model. And we also use LSTM as a baseline.

### Main results

Table 2 reports the overall performance of our model and baselines at one minute, one hour and one-day intervals. As shown in Table 2, it can be observed that:

1. Our proposed CVD-EDF significantly outperform other baseline models at all time intervals for both RMSE and MAE in steam turbine output power prediction. Compared with LSTM, CVD-EDF achieves an improvement of 32.2% RMSE and an improvement of 37.0% MAE at one-minute intervals. It proves the effectiveness of our proposed CVD-EDF and the reason for the improvement is that CVD-EDF takes into the vacuum degree information when predicting the output power of the steam turbine. It also demonstrates that taking into account the coupling relationship with the condenser is helpful for the output power prediction of the steam turbine.
2. When the time interval is longer, the advantages of our proposed CVD-EDF are more obvious. it achieves an improvement of 63.7% RMSE and an improvement of 68.8% MAE at one-day intervals compared to LSTM.
3. Regarding baseline models, LSTM achieves the best performance both on RMSE and MAE at one-minute and one-hour intervals while Decision tree regression performs best at one-hour intervals. Among the regression algorithms, ridge regression and linear regression have similar performance and they perform better than other algorithms, but they still fall short of our proposed CVD-EDF.

**Table 2:** Performance comparisons among several baselines at one minute, one hour and one-day intervals. The two metrics, RSME and MAE, are in megawatts (MW). (↓) represents “the smaller the better”.

Model Name	RMSE/MW(↓)	MAE/MW(↓)
**At one-minute intervals**		
Linear Regression	2.9658	2.3128
Ridge regression	2.8914	2.2440
LASSO regression	75.1732	60.4122
Elastic Net regression	75.1732	60.4122
Decision Tree regression	3.5969	2.5721
Xgboost	3.3797	2.5487
LSTM	2.5947	2.1656
**CVD-EDF**	**1.7597**	**1.3635**
**At one-hour intervals**		
Linear Regression	131.3898	102.3281
Ridge regression	126.0740	98.4166
LASSO regression	4406.8974	3550.1358
Elastic Net regression	4406.8974	3550.1358
Decision Tree regression	134.7658	93.8488
Xgboost	162.2692	121.7480
LSTM	124.2180	109.7862
**CVD-EDF**	**68.0847**	**53.6146**
**At one-day intervals**		
Linear Regression	2480.5151	2182.2465
Ridge regression	2355.0108	2071.8719
LASSO regression	86223.0892	63732.7707
Elastic Net regression	86223.0892	63732.7707
Decision Tree regression	1901.7868	1457.5911
Xgboost	3068.4372	2495.9845
LSTM	2666.4429	2539.8750
**CVD-EDF**	**968.0921**	**792.4782**

**Fig 3:**
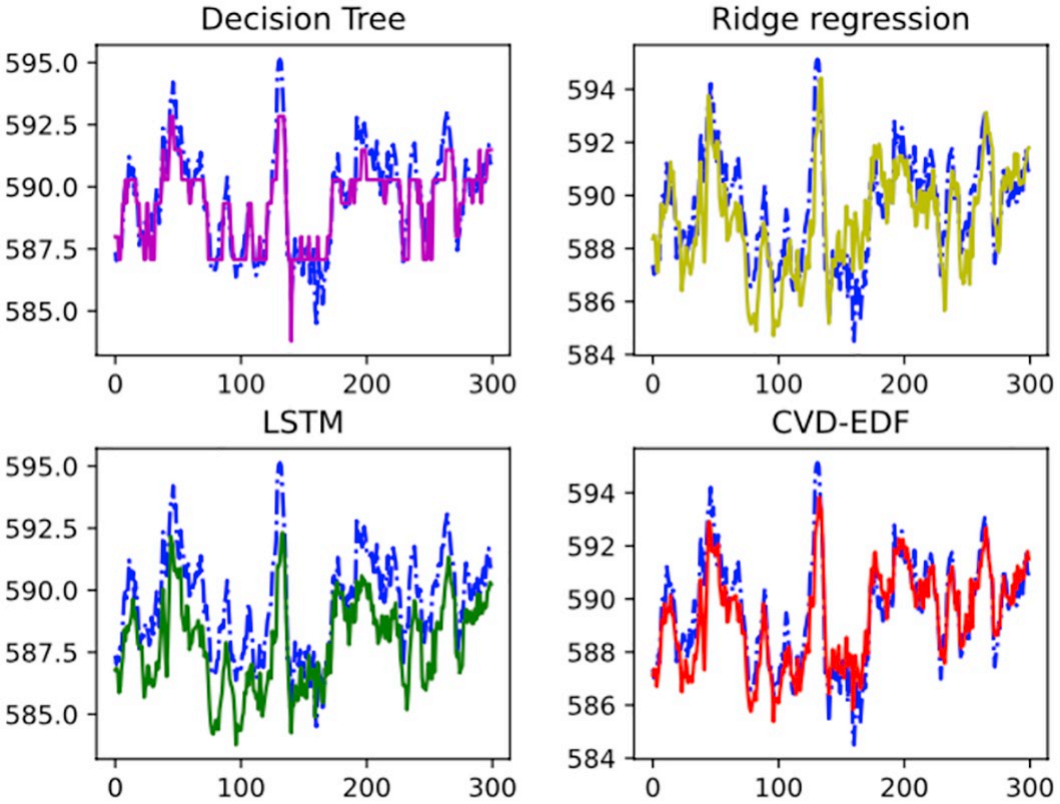
Timeline chart. The X-axis is the time step and the Y-axis is the predicted output power. The blue dot line corresponds to the actual output power.

Fig 3 is a timeline chart, it shows the prediction results of our proposed CVD-EDF for 300 consecutive moments samples in the test set at one-minute intervals. The blue line represents the actual steam turbine output power, while the red, yellow, green and purple lines represent the predicted steam turbine output power of our proposed CVD-EDF, ridge regression, LSTM and decision tree regression model respectively. It can be figured that:

1. our proposed CVD-EDF model is more consistent with the real steam turbine output power compared to decision tree regression, ridge regression and LSTM.
2. All the approaches have the ability to track the trend of the actual output power. However, the LSTM predictions are much lower than the actual steam turbine power almost all the time and the decision tree regression is not able to predict accurately during the time periods when the output power varies drastically. The ridge regression performs better than decision tree regression and LSTM, but the predictions are still inaccurate and more volatile. Our proposed CVD-EDF predictions are more accurate and informative.

### Ablation study

To further evaluate the effectiveness of each component, we conduct some ablation experiments on our model at one-minute intervals. We performed three ablation experiments:

- The input of the decoder does not contain the global features captured by CNN (i.e., CVD-EDF w/o CNN).
- The input of the decoder does not contain the local features captured by the attention mechanism (i.e., CVD-EDF w/o Attention).
- The input of the decoder is the historical data sequence of the steam turbine operating conditions without the local features and global features. The initial hidden states and cell states of the decoder LSTM are initialized to zero. Therefore, no condenser vacuum information is introduced from the encoder to the decoder and the model degenerates to a simple LSTM model. (i.e., CVD-EDF w/o Attention and CNN).

The results are shown in Table 3, and they can be summarized in the table:

1. Compared with CVD-EDF w/o Attention and CNN, CVD-EDF w/o Attention achieves an improvement of 24.6% RMSE and an improvement of 34.1% MAE while CVD-EDF w/o CNN achieves an improvement of 29.7% RMSE and an improvement of 34.4% MAE. It demonstrates that both the local feature and the global feature are beneficial for the steam turbine output power prediction.
2. Compared with CVD-EDF, the RMSE and MAE for CVD-EDF w/o CNN, CVD-EDF w/o Attention drop a lot. It means that the CNN and the attention mechanism are able to capture different aspects of vacuum degree information, and the model performs best when the two are combined.
3. Compared with CVD-EDF w/o Attention, CVD-EDF w/o CNN achieves an improvement of 6.9% RMSE and an improvement of 0.5% MAE. It means that the local features are relatively more important than the global features. The reason is that, at each step of the decoding process, the attention mechanism is able to dynamically determine different parts of the hidden states of the encoder. In contrast to global features, it is able to filter some irrelevant information and keep the most important information as input to the decoder.

**Table 3:** Ablation study results.

Model Name	RMSE/MW(↓)	MAE/MW(↓)
CVD-EDF w/o Attention	1.9573	1.4271
CVD-EDF w/o CNN	1.8231	1.4198
CVD-EDF w/o Attention and CNN	2.5947	2.1656
**CVD-EDF**	**1.7597**	**1.3635**

### Error analysis

**Fig 4:**
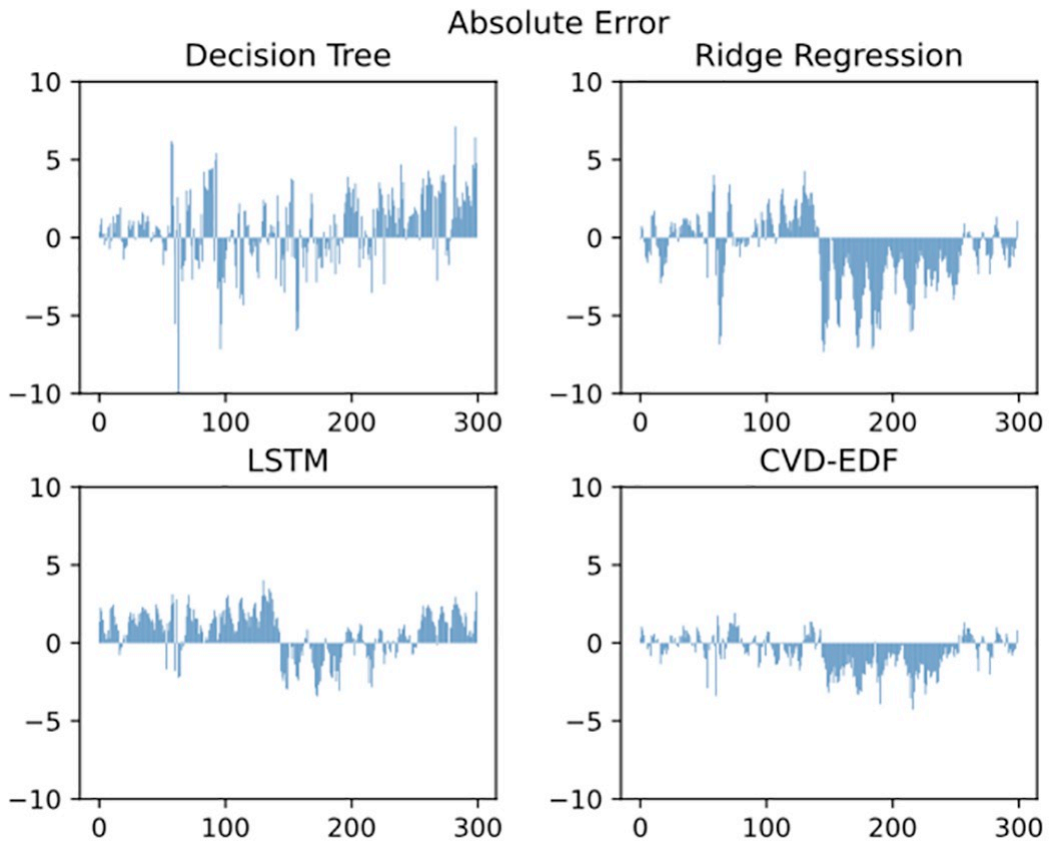
Steam turbine power prediction error comparison at one-minute intervals.

**Fig 5:**
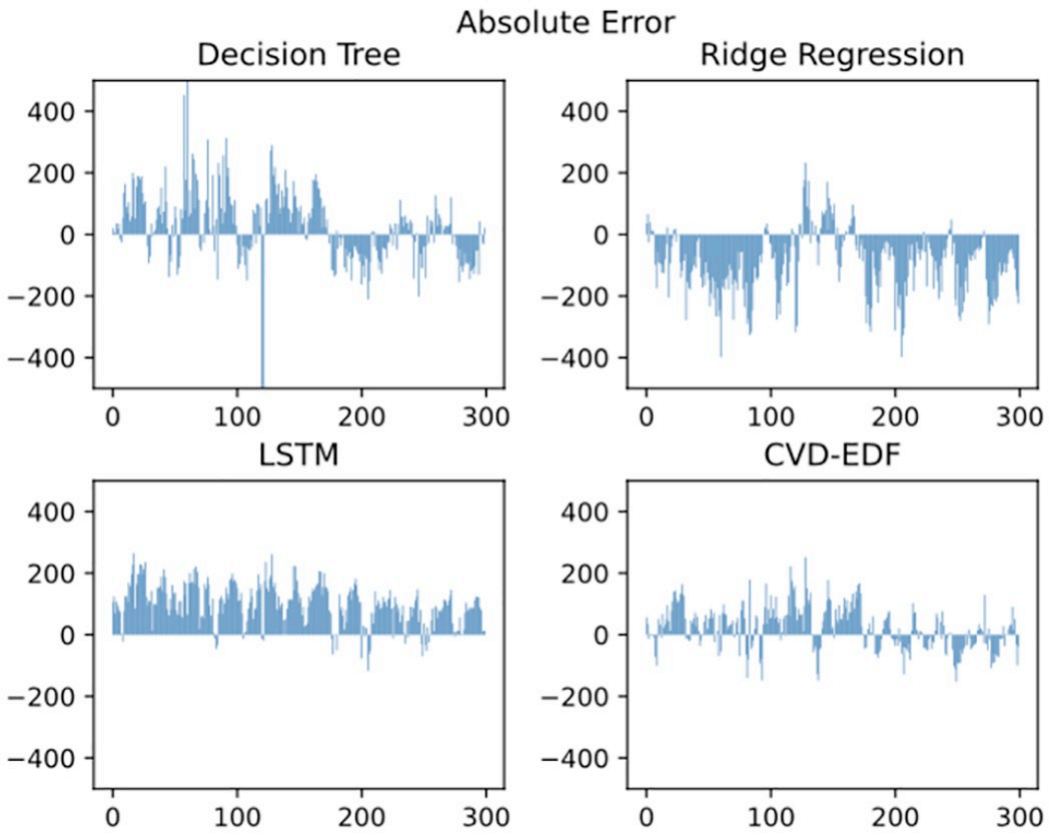
Steam turbine power prediction error comparison at one-hour intervals.

To further illustrate the performance of our approach, Fig 4 shows the prediction error of CVD-EDF for 300 test samples at one-minute intervals and Fig 5 shows the prediction error of CVD-EDF for 300 test samples at one-hour intervals, compared with decision tree regression, ridge regression and LSTM model. It can be observed that:

1. CVD-EDF effectively tracks the true steam turbine power trend and the error of CVD-EDF is smaller than other baselines both at one-minute intervals and one-hour intervals. The reason is that our approach leverages the encoder-decoder framework to introduce vacuum degree information in the steam turbine output power prediction.
2. LSTM, decision tree regression and ridge regression can track the trend of the actual steam turbine power. However, LSTM outputs much lower predictions than the real steam turbine output power almost all the time, while the ridge regression outputs much higher predictions than the real steam turbine output power most of the time. The decision tree regression predictions have large errors at some time steps. Therefore, the predictions of LSTM, decision tree regression and ridge regression are unreliable in a practical scenario.

## Conclusion

In this paper, we propose a novel approach for steam turbine power prediction based on the encode-decoder framework guided by the condenser vacuum degree (CVD-EDF), which for the first time explores the information on the coupling relationship between the steam turbine and the condenser. The condenser and steam turbine are modelled separately in the encoder part and the decoder part. In addition, a connection module which is composed of the attention mechanism and the CNN is proposed to capture the local and global information from the encoder. All of the information is introduced into the decoding process for accurate power prediction of the steam turbine. Experimental results on the real data collected from the power plant in Jiangsu Province of China show that the proposed method outperforms other competitive baselines. In the future, we will consider taking connected components of the thermal power generation system into account in the steam turbine power forecast, such as circulating water pumps.
