# Correction: Steam turbine power prediction based on encode-decoder framework guided by the condenser vacuum degree

**DOI:** 10.1371/journal.pone.0305366

**Published:** 2024-06-06

**Authors:** Yanning Lu, Yanzheng Xiang, Bo Chen, Haiyang Zhu, Junfeng Yue, Yawei Jin, Pengfei He, Yibo Zhao, Yingjie Zhu, Jiasheng Si, Deyu Zhou

In Fig 1, there is an error in one of the operator symbols on the Long Short-Term Memory (LSTM) neural network. It should have been a multiplication instead of addition. Please see the correct Fig 1 here.

**Fig 1:**
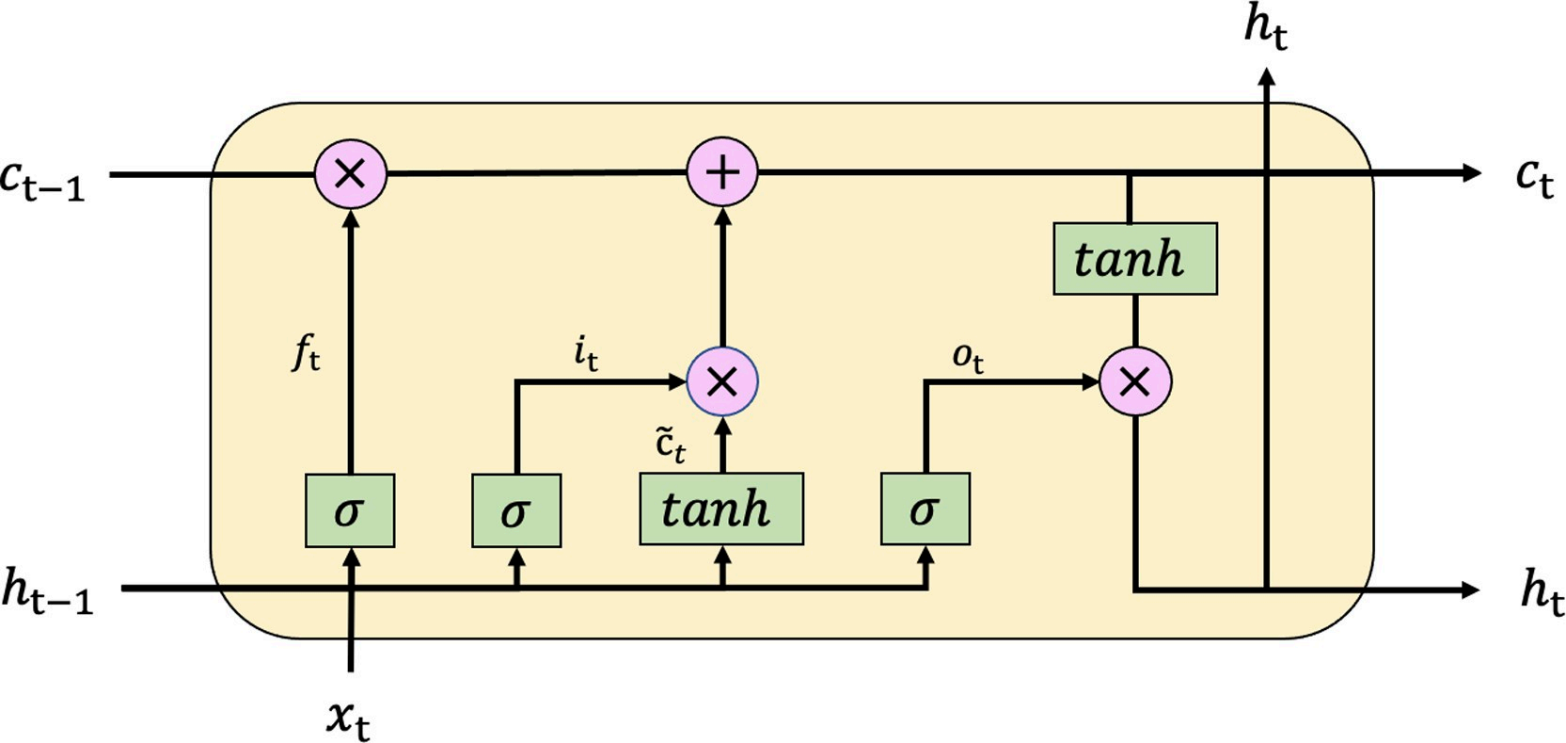
The architecture of LSTM.

Moreover, in the LSTM subsection of Preliminary, the Eq 3 is incorrect. The right bracket is missing. Please view the complete, correct equation here.


$${\tilde{c}}_{t}=tanh\left(W_{c}\cdot \left[h_{t-1},x_{t}\right]+b_{c}\right)$$ (3)
